# Chemopreventive Effects of *Onosma mutabilis* against Azoxymethane-Induced Colon Cancer in Rats via Amendment of Bax/Bcl-2 and NF-κB Signaling Pathways

**DOI:** 10.3390/cimb45020057

**Published:** 2023-01-18

**Authors:** Ahmed Aj. Jabbar, Ibrahim Abdel Aziz Ibrahim, Fuad O. Abdullah, Kareem Fattah Aziz, Abdullah R. Alzahrani, Mahmood Ameen Abdulla

**Affiliations:** 1 Department of Medical Laboratory Technology, Erbil Technical Health and Medical College, Erbil Polytechnic University, Erbil 44001, Iraq; 2 Department of Pharmacology and Toxicology, Faculty of Medicine, Umm Al-Qura University, Makkah 24382, Saudi Arabia; 3 Department of Chemistry, College of Science, Salahaddin University, Erbil 44001, Iraq; 4 Department of Pharmacognosy, Faculty of Pharmacy, Tishk International University, Erbil 44001, Iraq; 5 Department of Nursing, College of Nursing, Hawler Medical University, Erbil 44001, Iraq; 6 Department of Nursing, Faculty of Nursing, Tishk International University, Erbil 44001, Iraq; 7 Department of Medical Microbiology, College of Science, Cihan University-Erbil, Erbil 44001, Iraq

**Keywords:** colon cancer, Boraginaceae, chemoprevention, oxidative stress, histopathology

## Abstract

*Onosma* species (Boraginaceae) are well known as medicinal plants due to their wide range of pharmaceutical potential. The present study aims to investigate the anticancer (in vitro) and chemo-protective (in vivo) efficacies of *Onosma mutabilis* extract (OME) in the azoxymethane (AOM)-induced aberrant crypt foci (ACF) in rats. The in vitro antiproliferative effects of OME were determined on two human tumor cell lines (Caco-2 and HT-29) via MTT assay. The in vivo chemoprotective effects of OME were investigated by performing various biochemical analyses in serum and tissue homogenates of albino rats, along with determining oxidative stress biomarkers. Inflammatory biomarkers of colon, colonic gross morphology (by methylene blue), ACF formation, and colonic histopathology (H & E stain) were determined. The immunohistochemistry of colonic tissues was also assessed by Bax and Bcl-2 protein expression. The results showed that the antitumor activity of OME against Caco-2 and HT-29 colorectal cancer cells ranged between 22.28–36.55 µg/mL. OME supplementation caused a significant drop in the ACF values and improved the immunohistochemistry of the rats shown by up-regulation of Bax and down-regulation of Bcl-2 protein expressions. These outcomes reveal that *O. mutabilis* may have chemoprotective efficiency against AOM-induced colon cancer represented by the attenuation of ACF formation possibly through inhibition of free radicals, inflammation, and stimulation of the colon antioxidant armory (SOD, CAT, and GPx) and positive regulation of the Nrf2-Keap1 pathway.

## 1. Introduction

Colon cancer is considered one of the most common cancer types in males (726,450) and females (710,670) in the U.S. according to the latest reports from the American Cancer Society (ACS) [1]. Meanwhile, the rate of colon cancer-related death is expected to decline during the period 2015–2040, from 7.0 to 4.7 per 100,000 males, and from 5.3 to 3.2 per 100,000 females [2]. Surgical procedures and anticancer chemical drugs are two of the most common treatments for colon cancer [3]. The primary surgical removal of tumors is considered an effective technique if the cancer cell does not metastasize. Different forms of chemotherapy along with radiotherapy have also shown their effectiveness against cancers in the deep bowel wall or lymph nodes [4]. Different risk factors have been linked with the occurrence of colon cancer, including chronic intestinal inflammation, environmental mutagens, gut pathogens, unhealthy diet (high fatty diet), alcoholism, obesity, smoking, and lack of exercise. As the risk factors become more common among the global population, the search continues to find new pharmaceuticals with effective inhibitory potentials against colorectal cancer [5,6].

Colorectal cancer pathogenesis starts with the change of the intestinal epithelium into a polyp that could convert to a cancerous mode. Aberrant crypt foci (ACF), as prepolyp abnormalities, are identified as precursors to colorectal tumors in both animal laboratory models and humans. ACF was identified at an early stage as precancerous putative lesions in the colon of chemical-induced carcinoma-bearing rats. These colonic lesions are identified microscopically as crypts with oversized mucosa consisting of thick epithelial luminal and easily observed pericryptal areas [4]. The similarities in the gene property and appearance recognition of ACF in the colons of humans and animals have made ACF examination an easy tool to observe and identify the pathogenesis of colon cancer at early stages in research studies. ACF evaluation is considered an important procedure to examine the influences of possible chemoprotective agents on chemical-induced colon cancer rates [7,8]. On the other hand, an oxide of azoxymethane (AOM) is a well-known carcinogenic and ACF inducer that scientists widely use for cancer experiments [9].

Nutraceuticals play an important role as valuable sources for pharmaceutical production. Functional foods and medicinal plants have been widely used as effective treatments for various human health issues, including colon cancers [6]. Scientists have estimated that nearly 60% of cancer treatments originate from natural resources [10]. The bioactivities (antiproliferative and antioxidants) of natural metabolites have been explained through different mechanisms, including receptor targeting, reducing gene expression, and regulating enzymes of signal transduction pathways involved in cell proliferation (Bcl-2), apoptosis (p53), and drug resistance [11].

The genus *Onosma* is a group of well-known traditional medicinal plants that contain numerous phytochemicals offering different bioactivity against a wide range of moderate and severe human diseases [12,13]. The identified phytochemicals (rosmarinic acid, apigenin-7-O-β-glucoside, and globoidnan A) from *O. bourgaei* showed significant anticancer efficiency against human colorectal adenocarcinoma HT-29 and human mammary gland adenocarcinoma MCF-7 cells [14]. Furthermore, *O. bracteata Wall* was identified with different phytochemicals (1,2-benzene dicarboxylic acid and bis (2-methyl propyl) ester) and its aerial part extracts were found to be significant growth inhibitors of human lung carcinoma A549 cell, human osteosarcoma MG-63, and human neuroblastoma IMR-32 [15].

*O. mutabilis* is a newly identified species in Iraqi Kurdistan that contains various phytochemicals, naphthoquinones, phenolic, and phytosterols. Namely, phyto-constituents (especially Deoxy shikonin, β-sitosterol, Phenol, 2,4-bis (1,1-dimethyl ethyl)-, phosphite, and 24,25-Dihydroxycholecalciferol) were correlated with their increased antioxidant and in vitro antiproliferative potentials against prostate cancer cells (DU-145), human cervix carcinoma (Hep2c), and mammary cancer cells (MCF-7) as reported by the present author [16]. The present study investigates the anticancer (in vitro) potentials of OME against different colon cancer cells and the chemopreventive (in vivo) efficacy of OME in rats injected with AOM inducers of colon cancer by estimating the incidence of ACF and the evaluation of Bax/Bcl-2 protein expression.

## 2. Materials and Methods

### 2.1. Plant Extraction

The aerial parts of *O. mutabilis* were collected during spring 2022 on Safeen Mountain (GPS position: 36°311347” N, 44°427921” E). Authentication of the plant was carried out by botanist Prof. Dr. Abdullah Sh. Sardar (Department of Biology, Salahddin University, Erbil, Iraq). The specimen voucher has been deposited at the Education Salahaddin University Herbarium (ESUH) (Herbarium number: 7961). The plant organs were cleaned and put in a ventilated room for air-drying under shade at room temperature (20–25 °C) until fixed weight. OME was prepared through a maceration process; 200 g of aerial parts were separately soaked in 100 mL of methanol (99.99%) with occasional shaking in an ultrasonic bath for 20 min, then left in the same solvent at 30 °C for 24 h [17]. The procedure was repeated in triplicate for each plant organ. The methanolic mixtures were then filtered and the solvent was removed by rotary evaporator and the extracts were stored at + 4 °C until further analysis.

### 2.2. Cancer Cell

The human colorectal adenocarcinoma cells (Caco-2 and HT-29), and human dermal fibroblasts-adult (HDF-a) were purchased for the current in vitro cytotoxicity test. Doxorubicin (Dox) was used as a standard drug against selected cancerous cells. The Caco-2, HT-29, and HDF-a cells (Sigma-Aldrich Chemie GmbH) were purchased from the Merk cell Culture brand (Eschenstrasse 5, Taufkirchen, Germany). The 5 or 10% fetal bovine serum (FBS),100 lg/mL streptomycin, and 100 IU/mL penicillin (USA origin) were added to the culture media. Cells were put in an incubator with 5% CO_2_ at 37 °C for normal growth.

### 2.3. MTT Essay

Cancer cells were separated from the cell container by Trypsin-EDTA and then collected by centrifugation at 220 x g for 5 min. The cancer cells were re-suspended, and the cell density was estimated. The 96-well plate provided with 100 µL of growth media was used to seed tumor cells. The next day, 20 µL of various concentrations of plant extract was added to each well, while a 10% *v/v* DMSO was added as a blank. Forty-eight hours after OME addition, 10 µL of MTT (3- (4, 5-Dimethylthiazol-2-yl)-2, 5-diphenyltetrazolium bromide) solution was poured into the wells and then put in the incubator (4 h). The growth media were discarded, DMSO (100 uL) was added for the solubilization of formazan crystals, and the absorbency of the solution was measured at 595 nm [16,18].

### 2.4. Animal Study and Ethical Approval

The ethical guidelines governing the use of living animals for the experiments was strictly followed as stipulated by Iraqi animal rights and the study protocol was approved by the ECETHC (Ethics Committee of Erbil Technical Health College, Erbil, Iraq), which provided the ethical committee approval (Ref. No. 45 at 03-04-2022). Animal handling and care were in agreement with the guidance of the National Academy of Science for the Care and Use of Laboratory Animals [19].

### 2.5. Acute Toxicity Test

The toxicity experiment was performed to demonstrate the toxic and health effects of OME. The twelve male Sprague Dawley rats were equally grouped into the vehicle (normal control) and OME-treated groups based on the recommended guideline [20]. Food was taken away from rats for 24 h before the experiment and rats were only provided with water. A single dosage of 1000 mg/kg (low dose) and 2000 mg/kg (high dose) of OME were orally given to rats by gavage. Food was taken away for an additional 3 to 4 h and the animal observation took place every 30 min for 48 h after plant supplementation for checking the appearance of any change or abnormal behaviors. Based on the guidelines, if the OME-treated rats (2000 mg/kg) had 0–1 mortality, the safe dosage is expected to exceed 2000 mg/kg but if two or more rats died, then the given extract would be between 300–2000 mg/kg. The appearance of any signs and symptoms of toxicity, abnormal behavior, and death was noted over a time of 14 days. On the 15th day, the rats were given an overdose of anesthesia (xylazine and ketamine) and sacrificed. The blood samples were collected from intracardial puncture and then centrifuged (LC carousel, Roche, Germany) serum specimens were taken for biochemical investigation. The histology and serum biochemistry of experimental rats were investigated according to previously explained methods [21].

### 2.6. Experimental Design for the Chemoprotective Test of OME

The male Sprague Dawley rats (30 animals) were grouped randomly into five groups (6 animals for each): Group 1 (Normal control), Group 2 (Cancer control), Group 3 (Reference control), Group 4 (200 mg/kg OME), and Group 5 (400 mg/kg OME). The rats were given anesthesia before the injection of Azoxymethane (AOM) as an inducer for aberrant crypt foci (ACF) into rats is a common laboratory method for colon cancer investigation. The rats (besides the normal rat group) received 15 mg/kg of colon carcinogen (AOM) once a week for two weeks by subcutaneous injection. After that, Group A (Normal control) received Normal saline 15 mg/kg and then treated with 10% Tween 20 (5 mL/kg). Group B (Cancer control) was subcutaneously injected with AOM and then orally administered with distilled water. Group C (Reference control) was injected with AOM and intravenously injected with 35 mg/kg of 5-FU (5-fluorouracil). Group D (Low dose) was injected with AOM and administered OME (200 mg/kg) orally. Group E (High dose) was injected with AOM and administered OME (400 mg/kg) orally.

All rats had free access to food and water and the body weight of the rats was measured throughout the procedure. After two months, the rats were sacrificed and colon tissues were obtained for the histopathological examination. The tissue samples from colons were prepared by freezing with liquid nitrogen and homogenization.

### 2.7. Scoring of Aberrant Crypts

Rats were injected with anesthetic agents (ketamine and xylazine) in overdose and the obtained colon specimens were addressed with cold phosphate-buffered saline (PBS). The colon tissues were unwound longitudinally starting from the bottom (anus) to the rectum and then steeped in methylene blue dye (0.2%) to view and estimate the occurrence of aberrant crypt foci (ACF) by microscopic dissection. The crypt values for each specimen were determined and the ACF score was measured by calculating ACF in different focus.

### 2.8. Histological Examination of ACFs

Colon tissues were fixed with buffered formalin (10%) and then sectioned with a tissue-processing machine (Leica, Germany) before embedding in paraffin. The paraffin blocks were sectioned into 5- mm thicknesses and then stained with hematoxylin and eosin (H&E). The slides were observed for histological evaluation under a light microscope (Nikon, Tokyo, Japan).

### 2.9. Immunohistochemistry

The efficacy of OME on the expression of the Bax and Bcl-2 protein was evaluated by immunohistochemical technique [22]. Briefly, the tissue sections were deparaffinized and rehydrated and then immersed in sodium citrate buffer (10 mM) for 10 min, restoring their antigenicity. Tissue specimens were put in Tris-buffered saline for cooling before applying the ARK peroxidase kit (DAKO Denmark A/S, Glostrup, Denmark). The blocking of endogenous peroxidase was carried out by exposing them to the peroxidase solution for 5 min and then the samples were rinsed. The slides were incubated with biotinylated primary antibodies against Bax (1:100) and Bcl-2 (1:100) (Elabscience, Huston, TX, USA) for fifteen minutes and then treated with (exposed to) streptavidin-HRP for 30 min. The slides were stained with diaminobenzidine substrate chromogen and hematoxylin.

### 2.10. Antioxidants of Colon Homogenates

The obtained colon tissues were cleaned with ice-cold saline and the homogenate (10% *w/v*) was prepared with ice-cold phosphate buffer (50 mM, pH 7.4) containing a mixed mammalian protease inhibitor and centrifuged at 10,000× *g* for 30 min at 4 °C. The supernatant was separated to determine the activities of the catalase (CAT), superoxide dismutase (SOD), glutathione peroxidase (GPX) enzymes, and the content of malondialdehyde (MDA). The assay kits were purchased from Elabscience Co. (Houston, TX, USA).

### 2.11. Statistical Analysis

The determination was carried out in triplicate, and the results were shown as mean and standard error of the mean (mean ± SEM). The statistical significance of the results was determined by one-way analysis (ANOVA) and Tukey multiple comparisons of the SPSS using a statistical software package, version 24.0 for Windows. The *p* < 0.05 is considered a significant value between the groups.

## 3. Results

### 3.1. In Vitro Anticancer Effects

The results of the antiproliferative effects of OME on tumor cells are shown in Table 1. The results show significant anti-proliferation of OME against selected colorectal adenosarcoma (Caco-2 and HT-29) and normal dermal human fibroblasts (NHDF). The anticancer activity (*IC*_50_ values) of OME ranged between 22.28–36.55 µg/mL against Caco-2 and HT-29 cells. The anticancer (*IC*_50_) value of OME on NDHF was statistically higher (*IC*_50_: 173.26 μg/mL) than that of two colorectal cells. This indicates that OME has a significantly less cytotoxic effect on normal human fibroblast cells than on cancer cells. The outcome provides strong evidence for the inhibitory activities of OME toward the two colorectal cells: Caco-2 and HT-29.

### 3.2. Acute Toxicity

The present results of the oral toxicity of OME in the experimental rats showed a lack of undesired changes in the physiology or appearance of all tested rats. Rats treated with 1000 and 2000 mg/kg of OME showed normal behavior during and after 14 days of observational toxicity trial. The biochemical analysis of blood samples from experimental rats showed non-significant changes and all rats had values within the normal range for the liver and kidney function tests. Toxicity analysis revealed that OME-treated rats had similar data compared to normal controls.

**Figure 1:**
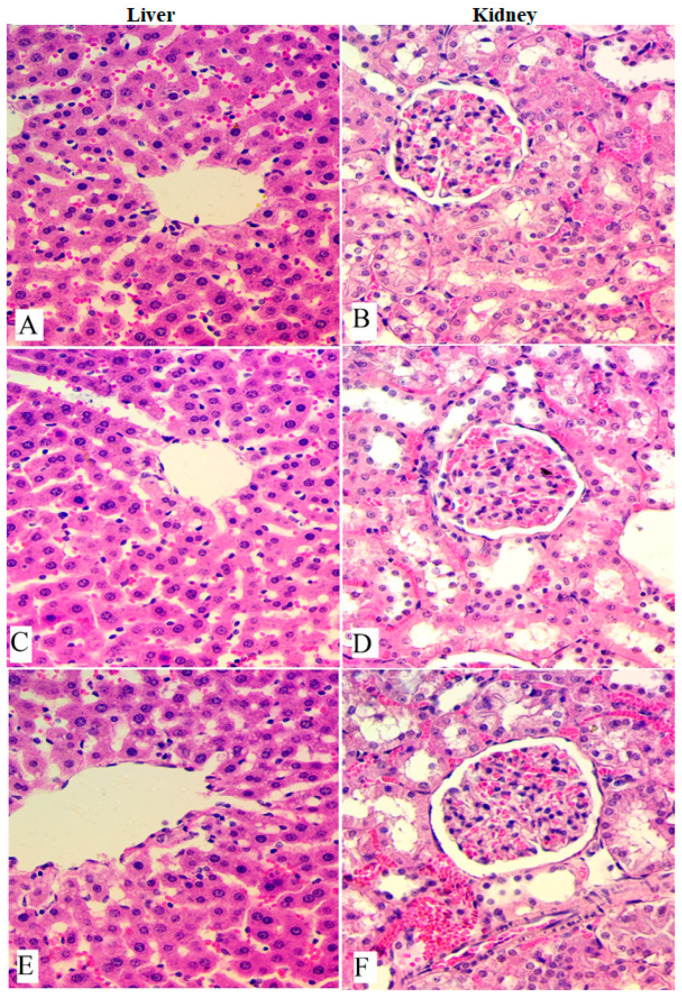
Histological views of liver and kidney obtained from rats in acute toxicity test. (**A**,**B**), Normal control; (**C**,**D**), rats administered 1000 mg/kg of OME; (**E**,**F**), rats treated with 2000 mg/kg of OME. Non-significant changes were observed between the experimental and normal rats according to microscopic views. H & E stain; magnification, 40×.

The histological examination of the liver and kidneys obtained from experimental rats was also carried out as presented in Figure 1. The microscopic observation revealed normal cellular structure and morphology of the liver and kidneys with the absence of any deformities in their color or appearance. These outcomes provide a scientific healthy dosage of OME and expect the toxic dosage of OME to exceed 2000 mg/kg (Table 2 and Figure 1).

### 3.3. In Vivo Anticancer Study

**Figure 2:**
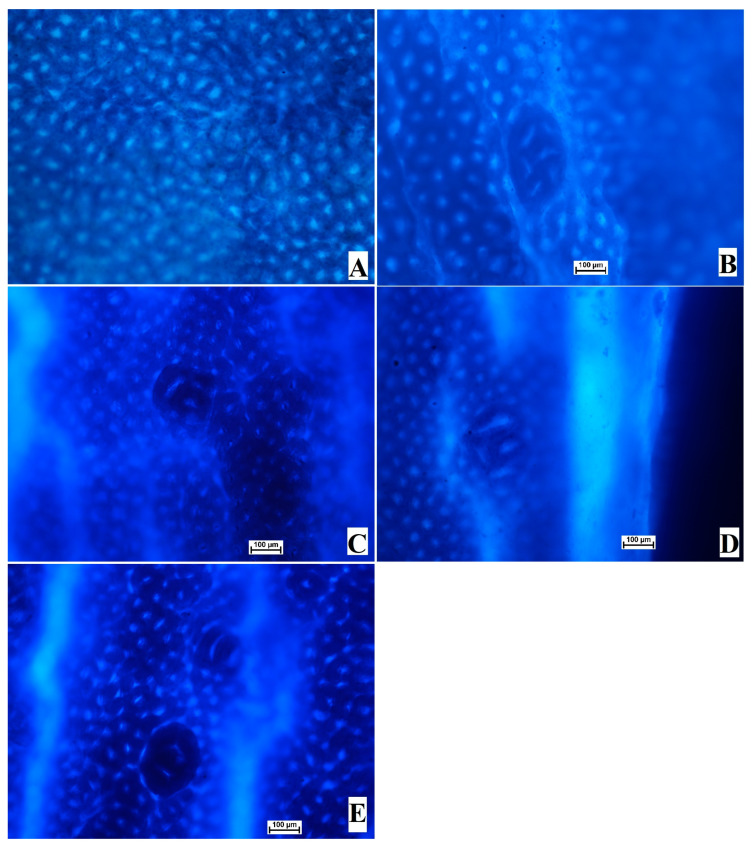
Influence of OME on the gross views of foci in the mucosal colon from experimental rats and with the methylene blue stain. (**A**), normal control; (**B**), cancer control (rats addressed with only AOM); (**C**), reference (rats administered 35 mg/kg of 5-FU); (**D**), low-dose OME (rats administered 200 mg/kg of OME); (**E**), High-dose OME (rats addressed with 400 mg/kg of OME). Aberrant crypts were distinguished from normal crypts by their larger size, their higher distance area between basal cell to the surface lumina, and their observable pericryptal region (methylene blue stain; 10× magnification).

The results of the current research showed the significant anticancer potential of OME in the azoxymethane-induced ACF in rats. The examination of ACF formation is considered an easy technique to determine the incidence of colon neoplasia. The colon tissues were analyzed to identify cancer after the methylene blue staining procedure. The topographical appearance of the colon tissue from experimental rats is presented in Figure 2. The normal control rats (A) showed the absence of ACF in their colon tissue stained with methylene blue (Figure 2 and Table 3).

**Figure 3:**
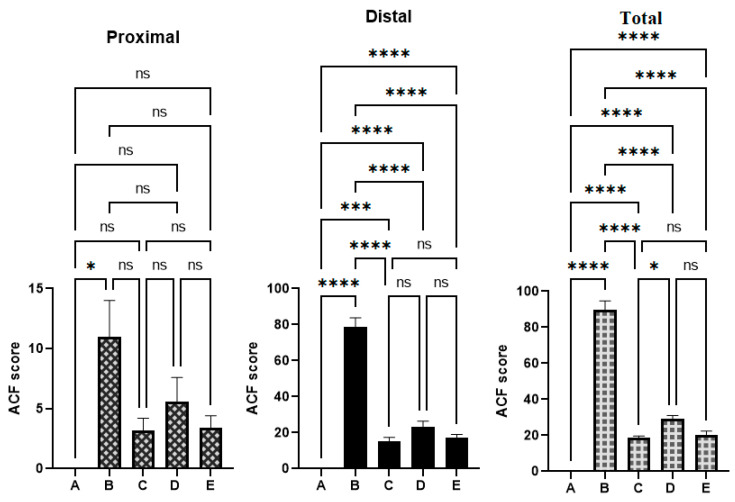
The ACF values were estimated from different parts of the colon from different rat groups. (A), normal control group; (B), cancer control group; (C), reference group; (D), rats supplemented with 200 mg/kg of OME; (E), rats treated with 400 mg/kg of OME. ns, non-significant; *, means significant at *p* < 0.05; ***, means significant at *p* < 0.001; ****, means significant at *p* < 0.0001.

As shown in the results presented in Figure 3 and Table 3, the cancer control rats showed significantly higher values of ACFs compared to the reference and OME-treated rats. OME-treated rats showed significant (*p* < 0.05) inhibition of foci formation in AOM-induced colonic ACF rats. The total number of the cancer control (B) crypts was significantly higher (89.7 ± 5) than that of 18.6 ± 1, 29.0 ± 2, and 20.4 ± 2 of reference (C), low-dose (D), and high-dose OME-treated rats (E), respectively. Rats treated with 400 mg/kg of OME showed non-significant differences in the number of foci compared to the reference group. The data analysis also showed the scatters of the ACF formation in the proximal and distal parts of the colons separated from the treated rats. The distal part of the colons showed more aggregation of ACF formation compared to the proximal part in all experimental groups (Figure 3).

The reference control (C) initiated up to 79.2% inhibition of ACF occurrence based on the obtained ACF values of the cancer control rats. Moreover, OME supplementation caused a reduction in AOM-induced ACF formation in a dose-dependent manner. Rats treated with 400 mg/kg of OME (E) showed a non-significant inhibition (77.25%) of the incidence of ACF compared to 79.2% of the reference group. 

The presented results show significant changes in the ACF formation between experimental and normal control groups; namely, both colon parts had significantly higher ACF values and ACF aggregations in cancer controls compared to other tested groups. OME treatments significantly lowered the ACF formation in the proximal and distal part of AOM-induced colon in rats (Figure 3).

### 3.4. Histological Analysis of Aberrant Crypt Foci

**Figure 4:**
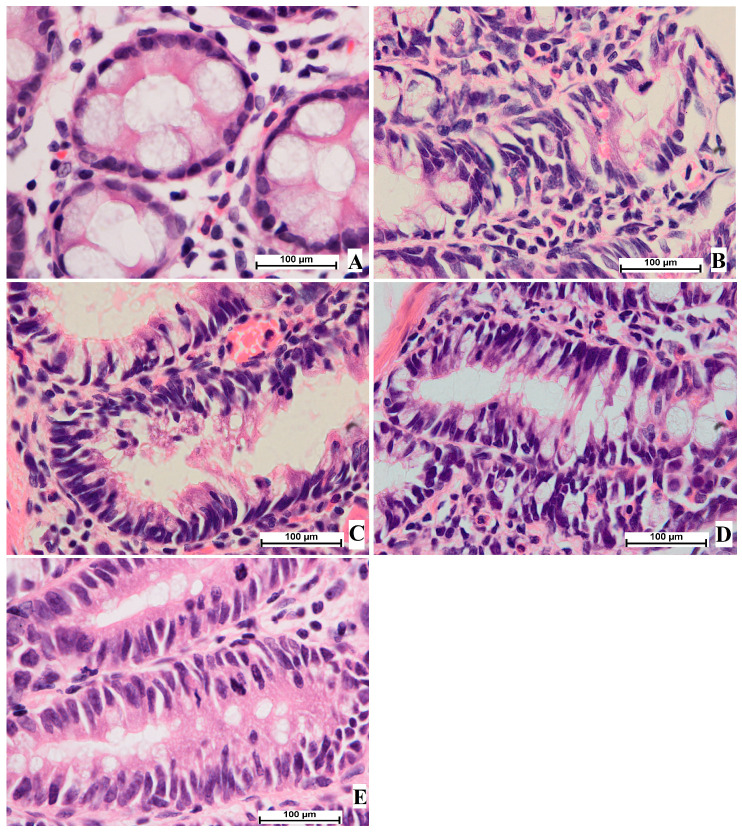
Histological study of colon tissue. (**A**), normal control group; (**B**), cancer control group; (**C**), reference group; (**D**), rats supplemented with 200 mg/kg of OME; (**E**), rats treated with 400 mg/kg of OME (H & E stain; 100× magnification).

The current histological results from staining colon tissue with hematoxylin and eosin revealed colon tissue damage with narrow lumens in epithelial cells, decreased cell polarity, elevated mitotic action, nucleus elongation, and absence of goblet cells in treated rats experiencing aberrant crypt foci. The microscopic views of the colon tissue showed circular cells with basal nuclei and normal mitosis. The results provide strong evidence of the efficacy of OME in the reduction of AOM-induced ACF production and pathological improvement in the mucosal linings of the colon in a dosage-dependent manner. The current study found that the number of cells with pathological changes decreased in rats treated with OME (D and E) or 5-fluorouracil (C), as shown in Figure 4.

### 3.5. The Bax and Bcl-2 Protein Expressions

**Figure 5:**
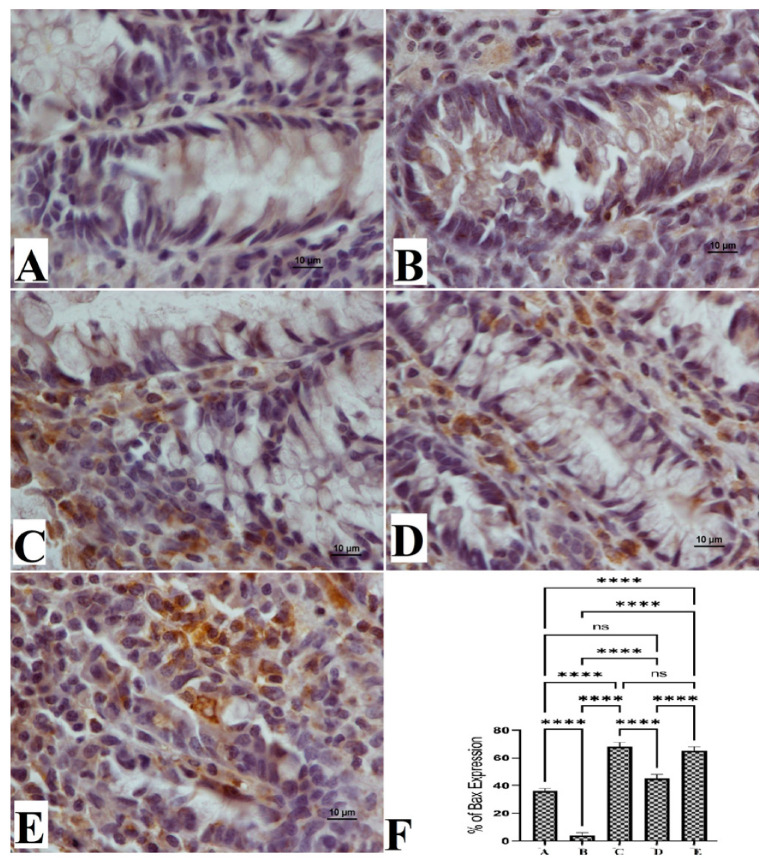
Histological views of colon tissue based on the Bax protein expression (**A**–**E**) and quantitative statistics (**F**) of Bax protein expression on colon tissue in induced foci in rats. Normal control rats (**A**) had normal mucosal structure and very weak expression of Bax protein (**F**). Colon cancer rats (**B**) had severe lesions in the mucosa and increased expression of Bax protein (**F**). Reference-treated rats (5-FU) (**C**) had mild mucosal colon tissue lesions and lower Bax protein expression (**F**). Rats treated with 200 mg/kg of OME (**D**) had moderate tissue damage and low Bax protein expression (**F**). Rats treated with 400 mg/kg OME (**E**) had mild mucosal lesions of colon tissue and lower Bax protein expression compared to cancer controls (**F**). (Bax stain, magnification 100×). Values are shown as means ± SEM. ns, non-significant; ****, means significant at *p* < 0.0001.

**Figure 6:**
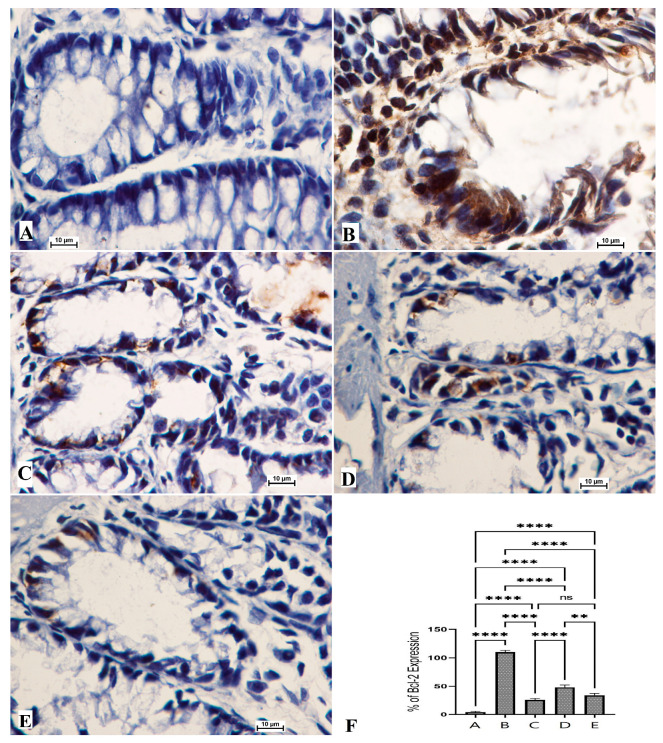
Histological views of colon tissue according to the Bcl-2 protein expression (**A**–**E**) and quantitative statistics (**F**) of Bcl-2 protein expression on colon tissue in induced foci in rats. Normal control rats (**A**) had normal colonic mucosa and very weak expression of Bcl-2 protein (**F**). Colon cancer rats (**B**) had severe lesions in the mucosa and increased expression of Bcl-2 protein (**F**). Reference rats (5-Fu) (**C**) had mild mucosal colon tissue lesions and lower Bcl-2 protein expression (**F**). Rats treated with 200 mg/kg of OME (**D**) had moderate tissue damage and low Bcl-2 protein expression (**F**). Rats addressed with 400 mg/kg OME (**E**) had mild mucosal lesions of colon tissue and lower Bcl-2 protein expression compared to cancer controls (**F**). (Bcl-2 stain, magnification 100×). Values are shown as means ± SEM. ns, non-significant; **, means significant at *p* < 0.01; ****, means significant at *p* < 0.0001.

The anticancer potentials of OME were also explored via the immunohistochemical examination of Bcl-2 and Bax protein expressions. The results showed that the cancer control group (B) exhibited notably lower immunostaining intensity for Bax expressions (Figure 5) and significantly higher staining intensity for Bcl-2 protein (Figure 6). Rats that received 200 mg/kg (D) and 400 mg/kg (E) showed significantly increased expression of the Bax protein and reduced expression of the Bcl-2 protein compared to cancer control rats, as shown in Figure 5.

The normal rat group (A) had normal color intensity in their colon tissue in the Bax immunostaining examination. The immunostaining intensity of Bax protein expressions of cancer control rats (B) was the lowest compared to all other groups. The reference standard group (C) was similar to those of OME-treated rats (Figure 5). Rats treated with OME had significantly upregulated expression in a dose-dependent manner (D, 200 mg/kg; E, 400 mg/kg). Based on the obtained data, experimental rats showed significant differences in their expression potentials of the Bcl-2 protein (Figure 6).

### 3.6. Antioxidative Enzyme Actions

**Figure 7:**
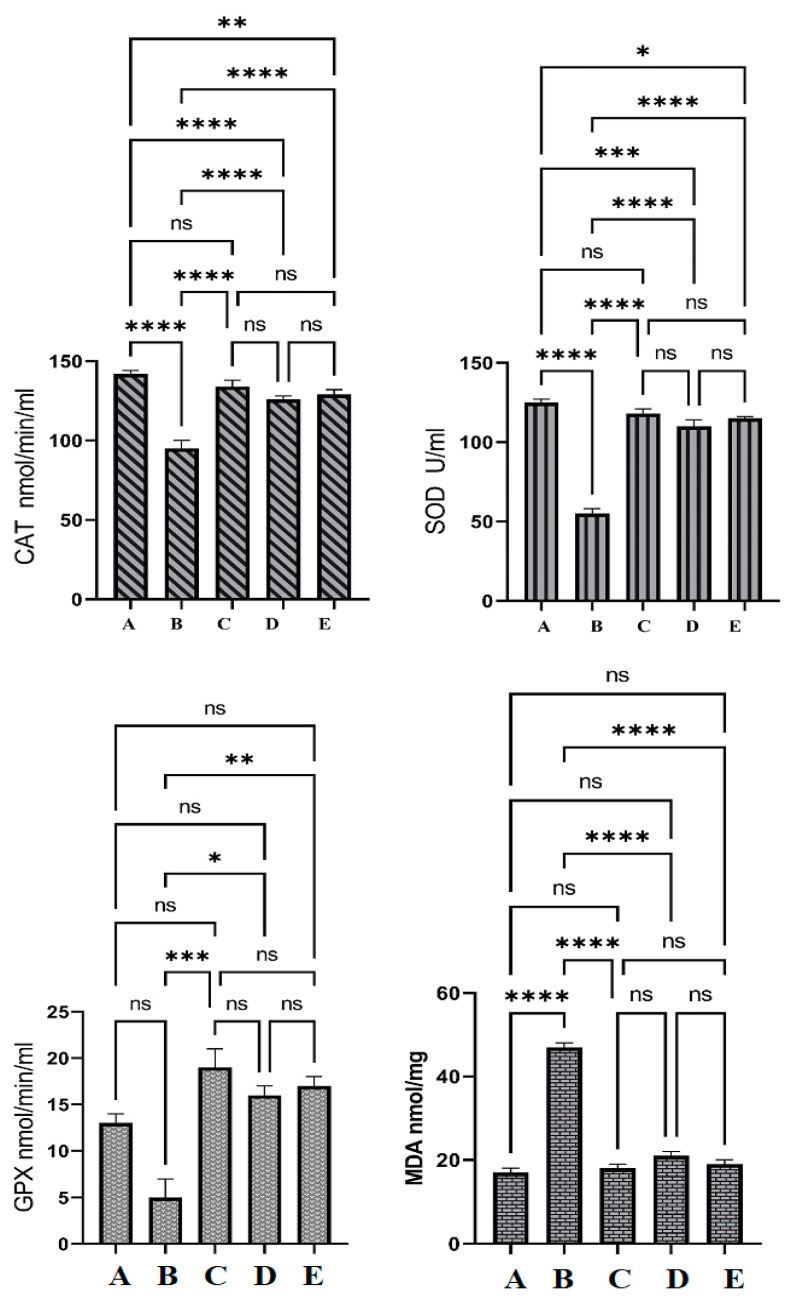
The antioxidant effects of OME in Azoxymethane (AOM) induced the aberrant crypt foci in rats. (A), normal control group; (B), cancer control group; (C), reference group; (D), rats supplemented with 200 mg/kg of OME; (E), rats treated with 400 mg/kg of OME. Cancer control had significantly lower endogenous antioxidant enzymes and higher lipid peroxidation values compared to all other groups. Non-significant changes in antioxidant measurements were observed between C, D, and E groups. ns, non-significant; *, means significant at *p* < 0.05; **, means significant at *p* < 0.01; ***, means significant at *p* < 0.001; ****, means significant at *p* < 0.0001.

The normal rat group (A) showed significant changes in the SOD and CAT levels in their colon tissue homogenates compared to all experimental groups and non-significant changes in the GPX and MDA levels compared to OME and 5-FU-treated rats. Rats treated only with Azoxymethane (AOM) (B) had significantly reduced levels of SOD, CAT, and GPX, and notably increased MDA contents in their colon tissue homogenates as shown in Figure 7. The endogenous antioxidant enzyme levels in colon tissue homogenates were not significant between OME-treated rats and reference drug (5-FU)-treated rats (C). The present results have shown significant up-regulation of SOD, CAT, and GPX in homogenized colon tissue in OME-treated rats (D and E) compared to those of the cancer controls. MDA was significantly reduced in OME-treated rats compared to that of cancer control rats, as shown in Figure 7.

## 4. Discussion

Medicinal plants have received more attention as alternative therapeutics and a raw source for modern drug production in the past decades. Previously, scientists have stated that about 60% of today’s anticancer remedies originate from natural plant-based products [23]. Researchers also have shown that herbal medicine possesses significant anticancer potentials against numerous cancer cells, breast cancer cells [24], colorectal cancers [25], and gastric and brain cancers [26]. The current study showed the significant in vitro potential of OME against two colon cancer (Caco-2 and H-29) cells. Recently, the cytotoxic effects of OME against mammary cancer (MCF-7), human cervix carcinoma Hep2c (HeLa), and prostate cancer (DU-145) have been shown by the present author. The significant biological activity of OME was linked with the plant’s antioxidant potential (1.12–2.33 mg/mL) possessed by its phytochemical contents, alkaloids (78.77%) and steroids (11.48%), especially 5,8-dihydroxy-2-(4-methylpent-3-enyl) naphthalene-1,4-dione, 3-O- Methyl-d-glucose, and β-Sitosterol as shown by the GC-MS analysis [16]. Similarly, researchers have shown experimental data results on the anticancer activity of *Onosma* species, *Onosma stellulata* [27], *Onosma bracteata* [15], and *Onosma bourgaei* [14]. Previously, scientists have shown the anticancer potential (against HCT 116 cell) of naphthoquinone (β-hydroxyisovaleryl shikonin) at the sub-G1 phase through its facilitating roles in the production of the reactive oxygen species (ROS) and inducing cancer cell apoptosis by caspase8/9 stimulation [28].

The results of acute toxicity testing present the safe dosage of this plant extract and suggest that the toxic dosage will be higher than 2000 mg/kg. Rats treated with 1000 and 2000 mg/kg did not indicate any toxic signs, with no significant changes in their serum biochemistry. Furthermore, the microscopic appearance of liver and kidney tissues obtained from OME-treated rats was comparable to that of normal control rats, with a lack of any visible tissue damage. This outcome confirmed the recently published study on the safe dosage of *O. mutabilis* [16].

The present study showed that rats treated with OME had significantly lower aggregation of foci formation in the distal and proximal parts of the colon compared to that of cancer control rats. The number of foci was significantly higher in the distal portion of the colon compared to those of the proximal portion. This outcome was inconsistent with previous research reports [14,29]. Foci formation was significantly reduced in rats treated with 200 mg/kg and 400 mg/kg of OME with an inhibition percentage of 67.5% and 77.25%, respectively. Similarly, several studies on other *Onosma* species and their phytoconstituents reported these species as reducing and preventive agents against colon cancer in rat models [13,14,15,27,30]. This outcome provides the promising potential of OME against AOM-induced colon cancer in rats.

The chemopreventive study of OME was also analyzed by immunohistochemical evaluation of two protein expressions in the colon tissue, Bcl-2 and Bax proteins. Studies have defined the Bcl-2 as an anti-apoptotic protein that blocks signals for apoptosis and Bax as the pro-apoptotic protein found in the outer membrane of mitochondria that regulates cell life or death through the stimulation of apoptosis by controlling the permeability of the mitochondria membranes [31]. A pro-apoptotic Bax protein has been considered as an inducer of apoptosis by dimerization and translocation into the outer membrane of the mitochondria, creating a route for further protein secretion, mainly cytochrome c., but this pathway could be reversed by anti-apoptotic proteins (Bcl-2) [32]. Thus, the balance between these two protein expressions is crucial for proper cellular function because any alteration in protein expression will directly change the mitochondrial route of apoptosis [33,34]. The current study showed that OME treatment leads to increased expression of Bax proteins and reduced expression of Bcl-2 proteins, which could also lead to activation of caspase-9 and caspase-3 (activation of immune system). The current immunohistochemical data showed significant colon tissue protection exerted by OME through up-regulation of Bax and down-regulation of Bcl-2 protein expression. Thus, OME-treated rats showed a smaller proliferation zone and labeling index, as the cells were removed from the growth cycle. Similar results were reported by several researchers, who showed the anticancer potentials of several herbal medicines via the upregulating of Bax protein and downregulating of Bcl-2 protein expression, which may induce apoptosis by mitochondrial mechanism [31,35,36].

The apoptosis pathway is affected by several factors that play a major role in the life or death of the cells and those factors are responsible for the outcomes of this process [37]. The colon cancer induced by AOM leads to a severe oxidative injury that leads to lipid peroxidation in colon tissue and the blood cells, which consequently stimulates more protein (albumin) secretion by the kidneys, increasing the blood urea level [38]. Harmony in the generation of ROS is crucial in cell function and the apoptosis pathway, and any disturbance or lack of ROS regulation could lead to significant cell organelle damage, reducing normal function, altering gene expression, cell metabolism, and cell arrest (death) [39]. The current study showed the significant potential of OME in balancing the ROS level. This biological action could be explained through the mechanism of scavenging free radicals and prevention of oxidative stress, which prevents colon tissue damage and initiates the colon tissue repair pathway. This bioactivity also could be correlated with its preventive role against ACF formation (induced by AOM) by its positive regulation of the antioxidant enzymes (CAT, SOD, and GPX) that prevent oxidative stress and strengthen the colon’s immune system.

The current work showed that rats treated with AOM showed severe oxidative damage, represented by significantly reduced levels of CAT, SOD, and GPX enzymes and increased lipid peroxidation shown by the upregulation of MDA levels. In contrast, plant supplementation (OME) led to a significant improvement in antioxidant status in AOM-induced AFC in rats. Accordingly, numerous scientists have shown the increased endogenous enzyme activities initiated by anticancer herbal products could be correlated with stimulating the pathways of chemoprotection therapy [40]. Furthermore, rats treated with OME had better oxidative status represented by significantly higher CAT, SOD, and GPX levels and decreased lipid peroxidation shown by lower MDA levels compared to cancer control rats. However, the difference was not significant between the treated groups because of OME-neutralizing effects on the toxic agents produced by the conversion of injected AOM into toxic metabolites. The in vitro antioxidant potential of OME has been reported by the present author, who has linked this bioactivity with its phytochemical content (napthoquinone, phenolics, and phytosterols) [16]. Previous studies have shown the antioxidant efficiency of numerous *Onosma* species, including *Onosma pulchra*, *Onosma ambigens*, and *Onosma echioides*, and they have linked this bioactivity with the phytochemical profile of *Onosma* species, napthoquinone, phenolics, and flavonoids, especially hesperidin, hyperoside, rosmarinic acid, apigenin 7-glucoside, pinoresinol, luteolin, apigenin chlorogenic acid, and luteolin 7-glucoside [13,41,42,43,44,45].

Oxidative stress is considered as one of the main risk factors of inflammation, which is mainly due to the irregularity of ROS production that consequently decreases the antioxidant enzymes required to eliminate the free radicals [46]. The stage 2 antioxidant-induced enzymes and cell protective genes (NAD (P)H quinone oxidoreductase 1 (NQO1), glutamate-cysteine ligase catalytic (GCLC), and heme oxygenase-1 (HO-1)) were stimulated by nuclear factor erythroid-2-related factor 2 (Nrf2), thus protecting the cells through a reduction in free radical formation and inflammation [47,48,49]. Nrf2 is combined with musculoaponeurotic fibrosarcoma (Maf) proteins in the nucleus and forms a cis-type bond with the antioxidants to initiate the transcriptional pathway. This pathway could be slowed by many genes, such as NQO1 and HO-1 as researchers explained [47]. Moreover, the NF-κB signaling action is suggested to reduce the antioxidant enzymes because of its suppressor effects on the Nrf2-Keap1 pathway by interacting the N-terminal region of p65 with Keap1 protein [50]. Therefore, the search for new compounds with anti-inflammatory Nrf2 signaling properties could enhance the cell’s antioxidant potential and increase the expression of cell protection genes (GCLC, HO-1, and NQO1) [51]. The present study revealed significant antioxidant action of OME that could be associated with its phytochemicals’ (napthoquinone, phenolics, and flavonoids) potential in the elevated expression of cellular defensive genes. Similarly, several studies have shown the potential of those chemicals in the positive regulation of the Nrf2-Keap1 pathway and the HO-1, NQO1, and GCLC genes [48,52,53,54].

## 5. Conclusions

The present study could be considered the first report describing the protective effect of OME on AOM-induced formation of foci in rats. The anticancer effects of OME on Caco-2 and HT-29 cells and the reduced formation of AOM-induced aberrant crypt foci (ACF) in rats were correlated with the positive impact of OME on the intracellular and extracellular mitochondrial mechanism. OME treatment leads to significantly increased antioxidant enzyme activity, reduced MDA contents, up-regulation of Bax, and down-regulation of Bcl-2 protein expressions (facilitating tumor cell apoptosis), which could be correlated with its phytoconstituents, including phenolic, flavonoid, alkaloid, and naphthoquinones. Thus, *O. mutabilis* could be an alternative natural source of chemical protectants against colorectal cancer, at least in its early stage, However, future studies could be considered to identify the exact phytoconstituents involved in this bioactivity by the bioassay-guided procedure for the explanation of the apoptosis-inducing effects of OME.

## Figures and Tables

**Table 1:** Anticancer activity *IC*_50_ (μg/mL) of *O.mutabilis* extract on Caco-2, HT-29, and L2OB after 24 h of treatment.

Cell	*IC*_50_ Values (μg/mL) ^1^	
	*O. mutabilis* MeOH Extract	DOX ^2^
Caco-2	22.28 ± 0.45 ^a^	0.83 ± 0.28
HT-29	36.55 ± 1.20 ^b^	0.62 ± 0.32
NHDF	173.26 ± 1.89 ^c^	1.22 ± 0.15

Keys: Caco-2 and HT-29, two colorectal adenosarcoma human cells. NHDF, normal dermal human fibroblasts. ^1^ Mean value ± SD of IC_50_ (μg/mL), inhibition concentration at which 50%. ^2^ DOX; Doxorubicin. Numbers with distinct superscript mean significance at *p* < 0.05.

**Table 2:** Influence of OME on the biochemistry of experimental rats in acute toxicity test.

Biochemical Parameters	Control	LD	HD
LFT			
Albumin g/L	32.2± 1.8 ^a^	33.1 ± 2.0 ^a^	33.1 ± 2.0 ^a^
Total bilirubin mmol/L	<2 ^a^	<2 ^a^	<2 ^a^
Alkaline phosphatase U/L	166.2 ± 24.4 ^a^	162.71 ± 23.2 ^a^	164.35 ± 36.8 ^a^
Alanine aminotransferase U/L	78.32 ± 11.8 ^a^	80.12 ± 14.1 ^a^	81.34 ± 12.2 ^a^
G-glutamyl transferase U/L	0.07 ± 0.5 ^a^	0.08 ± 0.4 ^a^	0.09 ± 0.3 ^a^
RFT			
Sodium mmol/L	139.2 ± 5.2 ^a^	142.5 ± 4.2 ^a^	143.4 ± 2.2 ^a^
Potassium mmol/L	4.9 ± 1.6 ^a^	5.1 ± 1.8 ^a^	5.7 ± 2.5 ^a^
Chloride mmol/L	102.5 ± 3.6 ^a^	104.08 ± 3.2 ^a^	105.12 ± 6.2 ^a^
Carbon Dioxide mmol/L	34.30 ± 1.9 ^a^	35.22 ± 1.3 ^a^	36.70 ± 2.3
Anion gap mmol/L	14.4 ± 1.4 ^a^	12.80 ± 1.7 ^b^	11.60 ± 3.5 ^b^
Urea mmol/L	5.80 ± 1.4 ^a^	6.10 ± 1.8 ^a^	6.8 ± 5.8 ^a^
Creatinine umol/L	31.20 ± 3.2 ^a^	29.10 ± 3.0 ^a^	28.52 ± 5.0 ^b^

Values are expressed as Mean ± standard error means (*n* = 6). Values with distinct superscript within the same rows indicate significance at *p* < 0.05. LFT, liver function tests; RFT, renal function tests. LD, rats treated with a low dose (1000 mg/kg) of OME; HD, rats treated with a high dose (2000 mg/kg) of OME.

**Table 3:** Inhibition potentials of OME on AOM-induced colonic ACF in rats.

Number of Foci Containing						
Groups	1 Crypt	2 Crypt	3 Crypt	≥4	Total	Inhibition %
				Crypt	ACF	
A	0	0	0	0	0	N/A
B	11.0 ± 3 ^c^	28.4 ± 4 ^c^	21.3 ± 1 ^c^	29.0 ± 1 ^c^	89.7 ± 5 ^c^	N/A
C	3.2 ± 1 ^a^	6.7 ± 2 ^a^	3.3 ± 2 ^a^	5.4 ± 1 ^a^	18.6 ± 1 ^a^	79.2 ^a^
D	5.6 ± 2 ^a^	8.3 ± 2 ^a^	7.0 ± 2 ^b^	8.1 ± 1 ^a^	29.0 ± 2 ^a^	67.5 ^b^
E	3.4 ± 1 ^a^	7.3 ± 1 ^a^	3.5 ± 2 ^a^	6.2 ± 2 ^a^	20.4 ± 2 ^a^	77.25 ^a^

Values are represented as mean ± SEM (*n* = 6). A, normal control; B, cancer control (rats addressed with only AOM); C, reference (rats administered 35 mg/kg of 5-FU); D, low-dose OME (rats administered 200 mg/kg of OME); E, high-dose OME (rats addressed with 400 mg/kg of OME). Values within the same column with distinct superscript mean significant difference at *p* > 0.05.

## Data Availability

Details regarding data supporting the reported results are available on request.
